# From Pilot to Scale, the 5 Year Growth of a Primary Care Pharmacist Model

**DOI:** 10.3390/pharmacy8030132

**Published:** 2020-07-30

**Authors:** Jordan Spillane, Erika Smith

**Affiliations:** Froedtert & The Medical College of Wisconsin, Wauwatosa, WI 53226, USA; erika.smith@froedtert.com

**Keywords:** ambulatory pharmacy, practice growth, academic medical center, innovative practice

## Abstract

This case report details the five year journey of implementing, growing and optimizing a primary care pharmacist model in the ambulatory clinic setting within a health system. There is published evidence supporting the numerous benefits of including pharmacists in the primary care medical team model. This case report provides information regarding evolution of practice, the pharmacists’ roles, justification and financial models for the pharmacist services, as well as lessons learned and determined conclusions.

## 1. Introduction

Over the past decade, health systems have undergone dramatic changes to meet the need for improved quality of care and outcomes. Health systems are now focused on the quadruple aim: enhancing patient experience, improving population health, reducing costs, and improving the work–life balance of health care providers [1]. In the ambulatory clinic setting, new strategies have stressed the importance of team-based, patient centered care that focuses not only on clinical outcomes but also on patient experience and financial implications [2]. Clinical pharmacists are in a unique position to be an essential member of this changing landscape and can provide effective collaboration to target the quadruple aim [3].

Multiple studies have shown the significant impact pharmacists can make to improve the quality of care and outcomes. In one large integrated health care system, pharmacists providing medication therapy management (MTM) services had an estimated return on investment (ROI) of $1.29 per $1 spent, while 95.3% of patients agreed or strongly agreed that their overall health had improved because of MTM [4]. Pharmacists have also shown benefit in several chronic disease states. For patients with atrial fibrillation, MTM services decreased emergency department visits, hospitalizations, and annual total care costs [5]. For patients with end stage renal disease, pharmacist interventions are estimated to save $3.98 for every $1 spent on pharmaceutical care [6]. For patients with uncomplicated mental health conditions, ambulatory care pharmacists supported an average decrease in PHQ-9 scores from 14.5 to 8.5 [7].

The American Society of Health System Pharmacists (ASHP) has the firm belief that pharmacists are vital in providing primary care [8]. With a 22% decline in primary care physicians, ASHP believes pharmacists can be utilized to improve access and continuity in care while contributing to chronic disease state management [8,9]. In addition, studies have shown that other medical professions are supportive of pharmacists in primary care settings. In a Likert survey (scale of 1–5) given to physicians and nurses who work with clinical pharmacists at an ambulatory cancer center, the response was overwhelmingly positive with median scores of 5 on questions such as the pharmacist had a positive impact and improved outcomes, the pharmacist allowed the clinic to run more efficiently, and a full time pharmacist in clinic would be valuable [10]. Throughout the country, health systems have implemented models that allow pharmacists to order laboratory tests, initiate or modify medications and educate patients with the goal of improving the quality of care that patients receive [8].

This case report describes one health system’s experience with the evolution of primary care pharmacist’s service scope, utilization, and outcomes from part-time pilot to scale. It is important to note the other contributing factors to the success of this journey including the health system growth, specifically a group practice physician strategy, evolving payer mix, and competitive landscape.

Froedtert Health comprises eastern Wisconsin’s only academic medical center, five hospitals, nearly 2000 physicians and more than 40 health centers and clinics. The health system represents the collaboration between Wisconsin’s largest multispecialty physician practice with two community-based physician groups. In the most recent fiscal year, outpatient visits exceeded 1.3 million, inpatient admissions were 52,855 and visits to the network physicians totaled 1,059,268.

## 2. Evolution of Primary Care Pharmacy

As the health system’s group practice strategy evolved so did the concept of leveraging the pharmacist. The ambulatory pharmacy department started at Froedtert Hospital, the academic medical center, in 1996 with a part time Anticoagulation Clinic created to address patient safety in relation to high risk medication management, as well as improve provider satisfaction. The model of the ambulatory pharmacy department has subsequently grown to support both specialty focused clinics and primary care. In this model, ambulatory clinic pharmacists develop collaborative practice agreements (CPA’s) that describe the disease states, medication classes, and labs that can be reviewed and ordered by the pharmacist. In Wisconsin, the law regarding the pharmacist’s scope of practice is broad and allows for physician delegation. In 2007, the organization identified a strategy to improve clinical education provided to its primary care affiliated clinics, while at the same time reducing the presence of pharmaceutical representatives in the clinic. The pharmacy department was asked to develop and support an unbiased provider education model around medications. This initiative started as twice-monthly educational sessions provided in person by pharmacists at four clinics. As the primary care site footprint grew from four to nine over a one-year period, this strategy evolved into virtual learning sessions (via teleconference) and the contracted FTE between primary care and pharmacy was re-evaluated. A pilot model of leveraging two part time pharmacists sharing time at four clinic sites was developed. This model included embedding the pharmacist physically at their clinic sites for ½ to 1 full day per week. The four largest sites were chosen to receive this support. Key outcome measures included drug therapy consults, cost savings, and therapeutic interchange initiatives. Over time, it also evolved to focusing on patients transitioning from the inpatient environment back to their primary care physician (PCP).

The model of part-time pharmacist support struggled in terms of proving concrete outcomes, as there was no specific disease state area of focus. It also proved to be challenging for the pharmacists to become a reliable member of the care team with such limited hours of support. Thus, the model evolved to a one-year pilot using one full time pharmacist splitting time 50/50 with two of the largest primary care clinics. A key component to the success of this second model was the pharmacist that was hired for this role had experience in establishing new services and working in a fully capitated health system supporting primary care clinical outcomes. The primary care pharmacist model quickly began to focus on outcome and cost based metrics with an emphasis on diabetes and inpatient transitions of care. Because of this focus, during a time when the health system was also starting its journey towards taking on financial risk with payer partners, the model was able to show demonstrable results for an outcome where there was already financial incentives. After a year, this pharmacist model was brought forward for executive leadership discussion and a second pharmacist FTE was approved. This pharmacist was positioned to focus on and support the newly formed Care Coordination department and shared risk goals. The concept of embedding a pharmacist within Care Coordination was felt to be a reasonable next iterative step towards scaling pharmacist support across the growing group practice locations.

In 2015, the health system formed a unified group practice of three legacy physician practices. This increased the number of disparate primary care sites from approximately 10 to 25. Senior leadership that led this new group practice identified the need for a pharmacy leader on their executive team and the pharmacy leader with the most ambulatory experience was chosen. This allowed for a deep shared understanding of the goals and strategies for both group practice success and ambulatory pharmacy expansion. The Chief Medical Officer (CMO) of the group practice also had a role in leading the population health strategies of the organization. By having a strong relationship with the CMO, the pharmacy leader was able to be at the forefront of developing population health strategies.

One area of focus was concerning patients with uncontrolled diabetes. Pharmacists providing diabetes care in ambulatory settings has been shown to decrease hemoglobin A1c, systolic blood pressure, and low-density lipoprotein cholesterol [11]. As a result, in 2016, a population health approach to managing uncontrolled type 2 diabetics (A1c > 9%) was formed and called the Ambulatory Diabetes Outreach Program (ADOP). This program included leveraging the two existing pharmacist FTE, as well as adding 1 FTE pharmacist and 1 FTE Certified Diabetes Educator (CDE) RN. This was the health system’s first step to proactive population based outreach and enrollment in a care team providing virtual support and disease state management to patients via telephone. The first patient population enrolled was the covered lives for which the health system had taken on some financial risk. The population health approach also included a strategy to integrate digital technologies to augment care by improving patient engagement in their healthcare. This strategy aligned with the health system’s investment in the formation of a new digital health accelerator company, and positioned ambulatory pharmacy as a key collaborator willing to work towards shared goals.

The primary care pharmacists were embedded in assigned clinics as much as possible with time present matching to clinic size, while the CDE RN focused support of the centralized care coordination department. Pharmacists also maintained provider-referred patients for other disease states including hypertension and polypharmacy. Having the specific population focus of the uncontrolled type 2 diabetic population (via the ADOP initiative) helped articulate the role and scope for the smaller sites with less historical exposure to pharmacist services.

Over the next 3 years, the demand for expansion and broadening of the primary care pharmacist model led to approval of an additional four FTE of primary care pharmacists. The program demonstrated a significant improvement in diabetes specific outcome goals, as well as broad patient and provider acceptance and demand.

## 3. Initial Lessons Learned

As alluded to above, having a pharmacy leader fully embedded in the group practice executive leadership team led to incredible insight and creative application of pharmacy support services beyond the typical primary care pharmacist role. This helped build trust and shared accountability in positioning the goal of a successful embedded primary care pharmacist program as something that multiple stakeholders had an interest in, such as group practice operations, population health/clinically integrated network, innovation and digital therapeutics. When opportunities arose, such as changes leading to access constraints in one market, the primary care pharmacist was thought of as a potential solution alongside advanced practice providers by the Chief Operating Officer (COO) of the group practice.

Consistency in the model of primary care pharmacist deployment is ultimately necessary. There are several options to consider for the primary care pharmacist model: centralized with no co-location at clinics, clinic attribution with minimal co-location, or site attribution with majority co-location. In each of the models, consistency must be maintained via a central pharmacy leader and intentional workload prioritization determined by pharmacy and senior clinic leadership. There are positives and negatives with each model (see Table 1). One key benefit of an embedded pharmacist model is that it allows for easier patient visit access and being potentially leveraged to avoid unnecessary physician visit utilization. The VA health system has optimized this model and subsequently changed both physician and patient expectations of whom and how their care is managed [12]. They have seen success with these changes resulting in increases in both number and scope of ambulatory pharmacists at VA systems [13]. One potential downside of embedding is that the pharmacist can be pulled into non-top of license broader care team work more easily. Within the centrally located model, the pharmacist can be successful with early adopter physicians who are open to collaboration with an extended care team member. Communication with various physicians can be more challenging in the centrally located model and can seem more onerous to the provider not having easy access face to face with the pharmacist. Lastly, splitting time amongst a high number of clinics and being primarily centrally located limits the pharmacist’s ability to be easily accessible for patient visits. 

In this health system, the model that has demonstrated the most success is clinic attributed embedded pharmacists aligned with an appropriate number of clinics based on patient volume/panel size, and with a clear population focus to align their services with the organization’s strategic goals. The embedded pharmacist allows for stronger relationship building with the providers and care teams, easier integration into patient care and better real time access for patient visits. Having these established relationships has also allowed for more facile development of new services, testing of new models, and quicker provider acceptance of patient care decisions made by the pharmacist.

## 4. Primary Care Pharmacist Roles

The pharmacist role in primary care and population health is to achieve valued outcomes for patients, as well as organizational goals. To accomplish this, the pharmacists provide care to patients that are referred by the physician or they proactively contact patients that match certain health metrics. The primary care pharmacists focus on both routine chronic disease state management services (diabetes, hypertension, polypharmacy/complex medication regimen review) and predetermined populations (i.e., diabetic patients with A1c >9, uncontrolled hypertensive patients on 2 or more blood pressure medications). This model recognizes the importance of the relationship between the patient and primary care physician, while also layering on services from the extended care team to selected uncontrolled populations without requiring the physician to determine that the patient needs additional support. The selected populations can be prioritized and evolve over time to align with patient, provider and organization need. These can include patients with select uncontrolled disease states, or there could be specific work done with a broader look at the patient’s various uncontrolled healthcare gaps, or include a medication specific focus such as safety initiatives or medication de-prescribing efforts.

Primary care pharmacists are leveraged as a resource to help in the ongoing education of providers and care teams. The primary care pharmacists write and circulate an electronic monthly newsletter of primary care focused topics, including guideline updates, primary care literature evaluations, and new drug approvals. Additionally, the pharmacists provide clinical presentations every quarter at the provider meetings across all the primary care health centers. This has been greatly appreciated by the providers and has helped remind all of the important role the pharmacists play as part of the clinic team. A primary care pharmacist is a member of the Ambulatory Therapeutics Committee and co-chaired a workgroup that focused on improving the safety and accuracy with administering vaccines across all clinics. The primary care pharmacists have been educated on the retail pharmacy services that the organization provides and have created positive working relationships with the retail pharmacists. This has helped the primary care pharmacists feel confident in recommending these services to their patients, which decreases the risk of polypharmacy, improves the rate of medication adherence and provides revenue to the organization.

## 5. Justification of Services

In order to maintain and optimize the primary care pharmacist model, it is very important to have access to data and communicate the benefits consistently to senior clinic leadership and physicians. In regards to the data, it is essential that an efficient and standardized workflow is created for the pharmacists to follow. This allows for the electronic health record to be optimized and provide data without requiring manual manipulation. A monthly dashboard of productivity and quality metrics was created and is shared with the primary care pharmacists on a monthly basis, and shared with the physicians and senior clinic leadership on a less frequent yet consistent basis. The dashboard can be drilled down to the individual pharmacist, which is shared by the pharmacy leader 1 on 1 with each pharmacist. This data helps the pharmacists feel confident in their clinical decisions and workflows, while also helping them determine what needs to be further refined to improve their individualized metrics. Based on these metrics and reviews of other primary care pharmacist models, a patient panel size was determined for each primary care pharmacist. This expectation was shared with the pharmacists and is reviewed on a monthly basis to hold each pharmacist accountable. Because the pharmacists are co-located at the clinics a majority of time, it is essential for the pharmacy leader to create a positive, virtual working relationship with each pharmacist. This typically involves face-to-face regular meetings at the start of the working relationship and demonstrated consistency with following-up on email communications. After the trusting relationship has been formed, having effective virtual meetings has proven to be successful. It is also essential that the pharmacist create strong working relationships with their providers, clinic staff and local clinic leadership. Finally, an important factor that should not be overlooked in ensuring success with this model is encouraging the physicians to talk to their patients about the role of the pharmacist in their care. The pharmacists have found that patients are more likely to agree to work with the primary care pharmacists and ultimately achieve better care outcomes when this warm hand off occurs.

## 6. Financial Models

There are several considerations in relation to the financial model to support expansion of primary care pharmacist services. The lack of provider status for pharmacists and subsequent inability to bill for clinical services provided by the pharmacists requires health systems to be creative to justify primary care pharmacist services. There are several options that allow for primary care pharmacists to provide clinical services without requiring the health system to incur the full expense of the FTE.

School of Pharmacy faculty funding support is an option that can provide a portion of a primary care pharmacist FTE to provide care in the ambulatory setting without requiring a significant financial investment from the organization. With this option, it is important to ensure the partnership with the School of Pharmacy allows for alignment of the faculty pharmacists model of care with what the organization has deemed as value added. Additionally, pharmacy residency training has expanded significantly, especially PGY2 ambulatory focused residencies, which has grown by 112% over the last five years [14]. By having PGY2 ambulatory focused residents, additional primary care pharmacy services can be provided at a significantly reduced financial rate.

There are other options that increase revenue for the organization to help with off-setting the expense of the primary care pharmacist FTE. Because pharmacists do not have provider status, it is essential to have positive working relationships with Compliance, Risk, IT and Finance representatives within the organization. This allows for optimization of reimbursement through compliant channels for the primary care pharmacists. In the non-hospital clinic space, pharmacists have minimal billing opportunities and primarily bill incident-to the provider. Billing incident-to requires direct supervision of the provider (must be located in the same clinic suite) at the time that the pharmacist is providing service to the patient and also has specific documentation requirements. While reimbursement is very minimal when billing incident-to in the professional setting, it is often helpful to demonstrate any incremental revenue, along with cost savings, to the organization. Payers may be interested in piloting a program where primary care pharmacists are paid for their patient encounters while being held accountable to shared clinical expectations and total cost of care reduction. If a strategic goal is to improve patient access to providers, pharmacists can provide certain visits to allow for physicians to focus on visits requiring diagnosis or complicated disease state management. Prescription revenue associated with utilization of the organization’s retail pharmacies is another way to tie actual dollars into support of pharmacy services. Other options focus on saving costs for the organization. Fully at risk organizations have a closely managed medication formulary for both in clinic administration as well as outpatient prescribing. Clinic based pharmacists have typically been very involved in the assessment and roll out of these initiatives leading to cost savings for their organizations [15,16]. And last but certainly not least, the continual growth of technology in support of efficient, evidence based care for populations will have significant impact on how the primary care practice of the future looks. Automation of appropriate patient care activities and stratification of populations that need additional management will continue to allow all care team members to operate more efficiently, including pharmacists.

Several of the above financial models have demonstrated success within the health system. The strong partnership with the Medical College of Wisconsin School of Pharmacy has allowed for additional FTE to provide pharmacy services in the primary care clinics, while also providing an excellent learning APPE experience to several learners. The health system has three PGY2 ambulatory pharmacy residents who complete a longitudinal primary care rotation, which allows for an entire additional 1.0 FTE to provide pharmacy services either through the residents’ staffing or by allowing additional capacity for the preceptors throughout the second half of the residency year. The majority of the primary care clinics are located in the professional space, so incident-to billing is utilized by the pharmacists when appropriate. Although this revenue is not significant, it is direct revenue correlated to the pharmacists’ services. When reviewing revenue from prescription capture, it is essential to have the data capabilities to drill down to the detailed level to determine how the primary care pharmacists are positively impacting this. The pharmacists have allowed for perceived increased access to the primary care providers by providing intensive focus on uncontrolled diabetes and optimizing hypertension medications without requiring PCP office visits.

## 7. Conclusions

Over the past 5 years, the primary care pharmacist team has grown from 1 FTE covering two primary care clinics to 7 FTE plus 3 PGY2 pharmacy residents covering all 25 primary care clinics. Determination for the appropriate scale for this innovative team is based on number of providers, provider panel sizes and the individual pharmacist’s panel size. A lot of work has occurred on implementing, maintaining and optimizing the organization’s current primary care pharmacist model. More work needs to continue in order to effectively grow this model. As previously stated, the ability to quantify how a pharmacist can improve physician access or increase their patient panel is important to the organization. An area of focus for the upcoming year is to create a definition of appropriate pharmacist utilization and calculate the associated financial outcomes related to that. It is important for pharmacy leadership to be aware of changes to the organization or payer landscape so that growth can occur in accordance with those changes. As quality metrics or shared risk models continue to grow in importance to the organization, it is essential to creatively think about how primary care pharmacists can impact those metrics effectively and efficiently.

It cannot be understated as to how important it is to share clinical data and successes of the primary care pharmacist model with the pharmacists, providers, clinic and senior leadership. The majority of the clinical data results for these primary care pharmacists is related to the uncontrolled type 2 diabetes patient population. All key stakeholders need to be consistently reminded of the positives and successes of the primary care pharmacist model. This will help with maintaining, as well as growing the model. The more people who understand what the pharmacists are capable of producing within clinic practice, the more success will be seen and pharmacists will be sought after to solve appropriate problems.

For some organizations, senior leaders and physicians, the primary care pharmacist model can be a new endeavor. The pharmacy leader will have a lot of heavy lifting to do while requesting and implementing this model. Relationships of the pharmacy team with others outside of pharmacy are key to the success of creating such a program. These relationships span all roles and disciplines across the organization. It is particularly helpful to understand what is most important to the key stakeholders so that data can center on that and be shared appropriately. It is also incredibly helpful to network with innovative ambulatory pharmacists and leaders outside of the organization to understand creative models and best practices. The pharmacy leader must demonstrate accountability by sharing both successes, as well as challenges and failures with the model. This case review documents the steps that were taken, primarily over a five year period, to request, implement, optimize, maintain and effectively plan for future growth of a primary care pharmacist model at a health system.

## Figures and Tables

**Table 1:** Pros/Cons with Pharmacist Models.

	Pros	Cons
Embedded Pharmacists Model	Easier patient visit access Can help avoid unnecessary physician visit utilization Proven to be effective and successful in several publications Better patient outcomes, patient satisfaction, better efficiency, etc. Streamlined face-to-face communication with providers Easier integration into care teams Improved transitions of care Can increase job satisfaction	Pharmacists must ensure their work is at the top of their education and training Logistics (i.e.,: physical space, clinic staff support) need to be determined
Centrally Located Model	Less disruptive to workflow within clinic Can streamline communication between pharmacists & leadership Improved equity amongst pharmacists	Communication with providers can be less efficient May take more time to create positive and trusting relationships with providers Workflows are necessary for appropriately scheduling pharmacist visits Model may be more reliant on the provider actively connecting the patient to the pharmacist

Conclusion: Ultimately, the embedded model was chosen due to its ability to allow for stronger relationship building with the care team, easier integration into providing care to patients, and better real time access for patient visits.
